# Autoclaving-treated germinated brown rice relieves hyperlipidemia by modulating gut microbiota in humans

**DOI:** 10.3389/fnut.2024.1403200

**Published:** 2024-05-17

**Authors:** Chuanying Ren, Bin Hong, Shan Zhang, Di Yuan, Junran Feng, Shan Shan, Jingyi Zhang, Lijun Guan, Ling Zhu, Shuwen Lu

**Affiliations:** ^1^Food Processing Research Institute, Heilongjiang Academy of Agricultural Sciences, Harbin, China; ^2^Heilongjiang Province Key Laboratory of Food Processing, Harbin, China; ^3^Heilongjiang Province Engineering Research Center of Whole Grain Nutritious Food, Harbin, China

**Keywords:** germinated brown rice, hyperlipidemia, gut microbiota, nutrition, functional food

## Abstract

**Introduction:**

Germinated brown rice is a functional food with a promising potential for alleviating metabolic diseases. This study aimed to explore the hypolipidemic effects of autoclaving-treated germinated brown rice (AGBR) and the underlying mechanisms involving gut microbiota.

**Methods:**

Dietary intervention with AGBR or polished rice (PR) was implemented in patients with hyperlipidemia for 3 months, and blood lipids were analyzed. Nutritional characteristics of AGBR and PR were measured and compared. Additionally, 16S rDNA sequencing was performed to reveal the differences in gut microbiota between the AGBR and PR groups.

**Results:**

AGBR relieves hyperlipidemia in patients, as evidenced by reduced levels of triglycerides, total cholesterol, low-density lipoprotein cholesterol, and apolipoprotein-B, and elevated levels of high-density lipoprotein cholesterol and apolipoprotein-A1. In terms of nutrition, AGBR had significantly higher concentrations of free amino acids (10/16 species), γ-aminobutyric acid, resistant starch, soluble dietary fiber, and flavonoids (11/13 species) than PR. In addition, higher microbial abundance, diversity, and uniformity were observed in the AGBR group than in the PR group. At the phylum level, AGBR reduced *Firmicutes*, *Proteobacteria*, *Desulfobacterota*, and *Synergistota*, and elevated *Bacteroidota* and *Verrucomicrobiota*. At the genus level, AGBR elevated *Bacteroides*, *Faecalibacterium*, *Dialister*, *Prevotella*, and *Bifidobacterium*, and reduced *Escherichia-Shigella*, *Blautia*, *Romboutsia*, and *Turicibacter*.

**Discussion:**

AGBR contributes to the remission of hyperlipidemia by modulating the gut microbiota.

## 1 Introduction

Hyperlipidemia is a common pathological condition in humans and is characterized by an abnormal elevation of blood lipids (1). High-fat diet, physical inactivity, and smoking are the main modifiable risk factors for hyperlipidemia that directly contribute to an increase in its prevalence worldwide (2). As lipid disorders are the leading causes of cardiovascular diseases, active treatment and prevention of hyperlipidemia are necessary in clinical practice (3).

Dietary habits are closely associated with the occurrence and development of hyperlipidemia. With the advancement of functional foods, a variety of crops have been developed that exhibit therapeutic potential for hyperlipidemia, such as oats (4), tartary buckwheat (5), black rice (6), and giant embryo rice (7). Brown rice is a whole grain with a bran layer and germ, and its sensory and nutritional qualities are improved by germination (8). Germinated brown rice (GBR) is not only rich in minerals, vitamins, essential amino acids, and dietary fibers, but also in various bioactive components, such as ferulic acid, γ-oryzanol, and γ-aminobutyric acid (GABA) (9). As a promising functional food, GBR is beneficial for the treatment of various human disorders, including hyperlipidemia, hypertension, diabetes, cardiovascular diseases, Alzheimer’s disease, and cancers (9). GBR has also been shown to contribute to the remission of hyperlipidemia by improving lipid metabolism (10–12). However, previous studies have mainly been conducted in animal models, and our knowledge of the hypolipidemic effects and mechanisms of GBR in the human body remains limited.

The gut microbiota is a complex system composed of large and diverse microbial communities (13). The gut microbiota plays a crucial role in maintaining human health, and its dysbiosis is associated with the progression of various human diseases, such as metabolic, gastrointestinal, cardiovascular, and neurodegenerative diseases (14). Notably, lipid metabolism *in vivo* can be affected by the gut microbiota, and hyperlipidemia is associated with obvious alterations in microbial composition (15). Previous studies have reported that many cereal foods are beneficial to the remission of hyperlipidemia via mediating gut microbiota homeostasis, such as oats (4), tartary buckwheat (5), black rice (6), red yeast rice (16), probiotics-fermented rice buckwheat (17), and highland barley (18). Zhao et al. reported that GBR relieves hyperglycemia, insulin resistance, and inflammation in high-fat diet-treated mice by increasing the abundance of probiotic bacteria such as *Lactobacillus gasseri* (19). However, how the gut microbiota participates in the action of GBR on hyperlipidemia in the human body remains unclear.

Autoclaving-treated GBR (AGBR) is a type of processed rice with high-quality taste, aroma, and nutrition (20). Our previous study showed that autoclaving improved the nutrition and storage of GBR by influencing the metabolites (21). In this study, AGBR and polished rice (PR) diets were implemented in patients with hyperlipidemia. After dietary intervention for 3 months, blood lipids and gut microbiota were analyzed and compared. We aimed to reveal the therapeutic potential of AGBR in hyperlipidemia and its mechanism of action involving the gut microbiota.

## 2 Materials and methods

### 2.1 Participants

A total of 56 patients with hyperlipidemia (25–65 years old; 32 men and 24 women) were included in the study. All enrolled patients had total cholesterol (TC) > 5.18 mmol/L and/or triglyceride (TG) > 1.70 mmol/L. The exclusion criteria were as follows: (1) pregnant or lactating women; (2) individuals with severe diseases involving the heart, liver, kidneys, hematopoietic system, or gastrointestinal system; (3) individuals with an allergic constitution or allergy to rice; (4) individuals with psychiatric disorders; and (5) excessive smoking or alcohol abuse.

### 2.2 Materials

Suijing 18, a Japonica cultivar (Suihua Branch of Heilongjiang Academy of Agricultural Sciences, Harbin, China), was used as the experimental material. Brown rice without husks and PR without a bran layer or germs were processed. To obtain AGBR, brown rice was germinated at 30°C and 95% humidity for 40 h, and then treated at 115°C for 20 min in a pressure-sterilizing pot. The PR and AGBR used for the intervention were semi-finished products in a vacuum package.

### 2.3 Dietary interventions

Before consumption, the product was steamed for 15 min. The enrolled patients were randomly divided into the PR and AGBR groups (*N* = 28 in each group). The PR or AGBR products were consumed for lunch (200 g) and dinner (120 g) as alternatives to the principal food. The dietary intervention lasted for 3 months. This study was approved by the Ethics Committee of Lianshui People’s Hospital Affiliated to Kanda College of Nanjing Medical University, and informed consent was obtained from all participants.

### 2.4 Measurement of biochemical parameters

Whole blood samples were collected from patients at baseline (0 month) and 3 months after dietary intervention. After centrifugation at 2500 rpm for 10 min at 4°C, the supernatants (serum samples) were collected and immediately used for measurements. Blood lipid parameters, including TG; TC, high-density lipoprotein cholesterol (HDL-c), low-density lipoprotein cholesterol (LDL-c), apolipoprotein-A1, and apolipoprotein-B, as well as liver function parameters, including alanine aminotransferase (ALT), aspartate aminotransferase (AST), and total bile acid (TBA) in the serum were measured by an automatic biochemical analyzer (GRT-3006, Glitter, Jinan, China) using specific kits (blood lipid parameters: Mlbio, Shanghai, China; liver function parameters: Jiancheng, Nanjing, China). Measurements were performed in triplicate for each patient.

### 2.5 Measurement of nutritional parameters

Nutritional characteristics of AGBR and PR were analyzed based on amino acids, dietary fiber, resistant starch, GABA, and flavonoids. Free amino acids (16 types) were measured using an amino acid analyzer. Soluble dietary fiber was measured using the enzyme weight method and resistant starch was measured by spectrophotometry (absorbance at 510 nm). GABA and flavonoids (13 types) were measured using a high-performance liquid chromatography-O-Orbitrap-mass spectrometry system (Vanquish, Thermo Fisher Scientific, Waltham, MA, United States), and the data were acquired using Xcalibur 4.1 and processed using TraceFinder 4.1 (Thermo Fisher Scientific). Measurements were performed in triplicate for each patient.

### 2.6 16S rDNA sequencing

Fecal samples were collected at baseline (0 month) and 3 months after dietary intervention, and were stored in sterile tubes at −80°C until further use (PR, *N* = 19; AGBR, *N* = 18). Genomic DNA was extracted from fecal samples of patients with hyperlipidemia using the CTAB method. The V3–V4 regions of 16S rDNAs were amplified by PCR, and the products were purified. Subsequently, a library was constructed using the NEBNext Ultra II DNA Library Prep Kit (New England Biolabs, Ipswich, MA, United States). The established library was quantified using a Qubit 2.0 fluorometer (Life Technologies, Carlsbad, CA, United States), assessed using an Agilent 2100 Bioanalyzer system (Agilent, Santa Clara, CA, United States), and finally sequenced using NovaSeq 6000 (Illumina, San Diego, CA, United States).

### 2.7 Microbial analyses

Reads were assigned to samples and merged into raw tags using FLASH after removing barcodes and primer sequences. Effective tags were obtained by screening high-quality clean tags using fastp, and filtering chimeras using Usearch. Effective tags were then denoised using DADA2 to obtain amplicon sequence variants (ASVs). The ASV species information was annotated using QIIME2 based on the Silva138.1 database. Alpha (goods_coverage, chao1, observed_utos, Shannon, Simpson, and pielou_e) and beta diversities (unweighted and weighted PCoA) were analyzed using QIIME2.

### 2.8 Statistical analyses

Quantitative data were expressed as mean ± standard deviation (SD) and were statistically analyzed using SPSS (version 17.0, SPSS Inc., Chicago, IL, United States). The differences between the PR and AGBR groups were determined using independent *t* test. Differences were considered statistically significant for *p* < 0.05.

## 3 Results

### 3.1 Dietary intervention of AGBR reduces blood lipids in patients with hyperlipidemia

The potential of AGBR in lowering blood lipid levels was evaluated in patients with hyperlipidemia. As shown in Table 1, dietary intervention with AGBR for 3 months significantly reduced TG, TC, and LDL-c levels in patients with hyperlipidemia (*p* < 0.05). Patients with hyperlipidemia who consumed AGBR for 3 months also exhibited significantly higher HDL-c levels than those who consumed PR (*p* < 0.01). The levels of apolipoproteins (A1 and B) were measured to determine the effects of AGBR on hyperlipidemia. As expected, 3 months of AGBR diet significantly elevated apolipoprotein-A1 levels; however, it reduced apolipoprotein-B levels in patients with hyperlipidemia (*p* < 0.05) (Table 1).

**Table 1:** Blood lipids in patients with hyperlipidemia who consumed polished rice (PR) or autoclaving-treated germinated brown rice (AGBR).

Parameters	Baseline (0 month)			3 months		
	PR	AGBR	*p*-value	PR	AGBR	*p*-value
TG (mmol/L)	1.971 ± 0.885	1.936 ± 0.642	0.866	2.358 ± 1.046	1.723 ± 0.495	0.005*
TC (mmol/L)	4.845 ± 1.018	4.786 ± 1.170	0.841	5.077 ± 1.014	4.289 ± 0.961	0.004*
HDL-c (mmol/L)	1.065 ± 0.162	1.082 ± 0.186	0.717	1.038 ± 0.265	1.362 ± 0.271	<0.001*
LDL-c (mmol/L)	2.966 ± 0.829	3.045 ± 0.744	0.709	3.392 ± 0.711	2.809 ± 0.801	0.006*
Apolipoprotein-A1 (g/L)	0.980 ± 0.217	1.028 ± 0.227	0.422	1.101 ± 0.232	1.259 ± 0.186	0.007*
Apolipoprotein-B (g/L)	0.819 ± 0.253	0.846 ± 0.286	0.710	0.975 ± 0.237	0.787 ± 0.248	0.005*

TG, Triglyceride; TC, Total cholesterol; HDL-c, High-density lipoprotein cholesterol; LDL-c, Low-density lipoprotein cholesterol. *Significantly different between PR and AGBR.

### 3.2 Dietary intervention of AGBR does not affect liver function in patients with hyperlipidemia

As liver function is closely associated with lipid metabolism, some representative parameters were measured in patients with hyperlipidemia who consumed PR or AGBR. At baseline (0 month), no significant differences in liver function parameters were observed between the PR and AGBR groups. After 3 months of dietary intervention, ALT, AST, and TBA levels were slightly decreased in patients with hyperlipidemia who consumed AGBR compared to those who consumed PR; however, the differences were not significant (Table 2).

**Table 2:** Liver function in patients with hyperlipidemia who consumed polished rice (PR) or autoclaving-treated germinated brown rice (AGBR).

Parameters	Baseline (0 month)			3 months		
	PR	AGBR	*p*-value	PR	AGBR	*p*-value
ALT (U/L)	27.94 ± 13.39	28.53 ± 14.70	0.876	25.47 ± 12.45	22.47 ± 9.56	1.011
AST (U/L)	24.82 ± 8.99	25.29 ± 9.95	0.854	23.12 ± 9.30	21.71 ± 5.65	0.686
TBA (μmol/L)	4.15 ± 2.41	4.03 ± 2.41	0.853	4.02 ± 1.93	3.54 ± 1.44	0.296

ALT, Alanine aminotransferase; AST, Aspartate aminotransferase; TBA, Total bile acid.

### 3.3 Nutritional characteristics of AGBR

The nutritional characteristics of AGBR and PR were compared to reveal the underlying mechanisms by which AGBR alleviates hyperlipidemia. As shown in Table 3, the levels of most free amino acids (10/16) in AGBR were higher than those in PR (*p* < 0.05). Compared with PR, AGBR also exhibited significantly higher concentrations of GABA, resistant starch, and soluble dietary fiber (*p* < 0.05). Additionally, the concentrations of 11/13 flavonoids were significantly higher in AGBR than in PR (*p* < 0.05).

**Table 3:** Comparisons of nutritional parameters between polished rice (PR) and autoclaving-treated germinated brown rice (AGBR).

Parameters	PR	AGBR	*p*-value	Parameters	PR	AGBR	*p*-value
Free amino acids (g/100 g)				GABA (mg/100 g)	0.31 ± 0.15	34.53 ± 3.09	<0.001*
Total	5.23 ± 0.04	8.31 ± 0.08	<0.001*	Resistant starch (g/100 g)	0.30 ± 0.02	2.85 ± 0.07	<0.001*
Asparagine	0.52 ± 0.02	0.51 ± 0.01	0.482	Soluble dietary fiber (g/100 g)	0.26 ± 0.01	0.65 ± 0.02	<0.001*
Threonine	0.20 ± 0.01	0.21 ± 0.01	0.288	Flavonoids (ng/100 mg)			
Serine	0.23 ± 0.01	0.31 ± 0.01	0.001*	Catechin	0.13 ± 0.01	0.20 ± 0.01	0.001*
Glutamic acid	0.97 ± 0.02	0.94 ± 0.02	0.140	Epicatechin	0.12 ± 0.02	0.19 ± 0.01	0.006*
Glycine	0.27 ± 0.01	0.28 ± 0.01	0.288	Rutin	0.62 ± 0.07	1.29 ± 0.03	<0.001*
Alanine	0.27 ± 0.01	0.28 ± 0.01	0.288	Vitexin	0.45 ± 0.10	4.60 ± 0.03	<0.001*
Valine	0.37 ± 0.01	0.42 ± 0.01	0.004*	Quercetin 3-β-D-glucoside	0.23 ± 0.02	0.26 ± 0.02	0.140
Methionine	0.19 ± 0.01	0.27 ± 0.01	0.008*	(+)-Dihydroquercetin	<0.01	0.31 ± 0.01	<0.001*
Isoleucine	0.22 ± 0.01	0.27 ± 0.01	0.004*	(+)-Dihydrokaempferol	<0.01	0.09 ± 0.01	<0.001*
Leucine	0.43 ± 0.01	0.52 ± 0.01	<0.001*	Luteolin	0.17 ± 0.02	0.84 ± 0.03	<0.001*
Tyrosine	0.12 ± 0.01	0.14 ± 0.03	0.335	Quercetin	0.08 ± 0.06	1.05 ± 0.13	<0.001*
Phenylalanine	0.36 ± 0.01	0.90 ± 0.03	<0.001*	Apigenin	0.02 ± 0.01	0.50 ± 0.01	<0.001*
Lysine	0.20 ± 0.01	0.28 ± 0.01	0.001*	Naringenin	0.04 ± 0.04	0.33 ± 0.01	<0.001*
Histidine	0.14 ± 0.01	0.21 ± 0.01	0.006*	Kaempferol	0.05 ± 0.02	0.08 ± 0.01	0.081
Argnine	0.42 ± 0.01	1.94 ± 0.02	<0.001*	Isorhamnetin	<0.01	0.67 ± 0.07	<0.001*
Proline	0.19 ± 0.01	0.26 ± 0.01	0.001*				

GABA, γ-aminobutyric acid. *Significantly different between PR and AGBR.

### 3.4 Dietary intervention with AGBR changes gut microbiota in patients with hyperlipidemia

**Figure 1:**
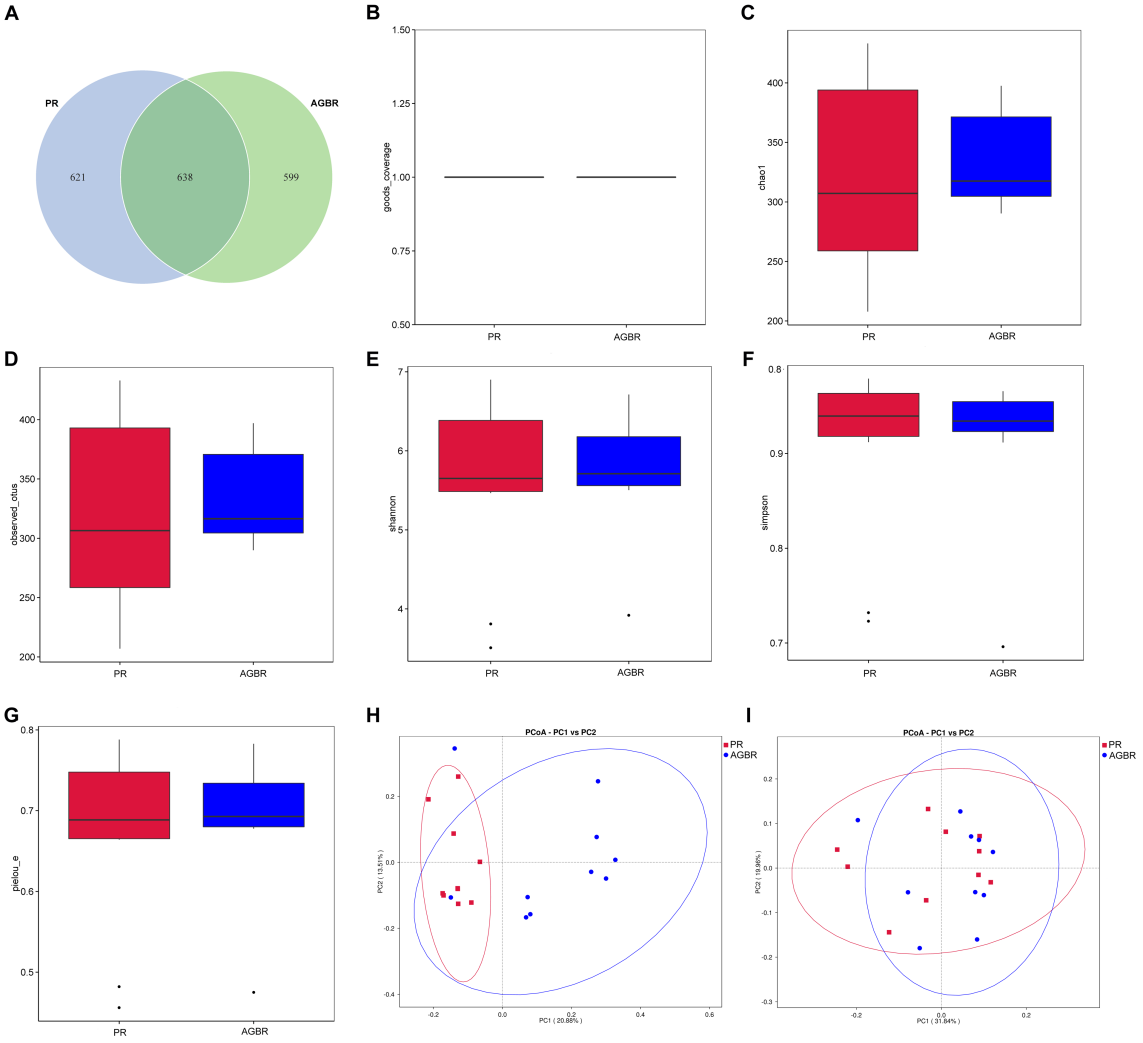
The distribution of gut microbiota in patients with hyperlipidemia receiving AGBR or PR diets. **(A)** Venn diagram. **(B)** Goods_coverage. **(C)** Chao1. **(D)** Observed_utos. **(E)** Shannon. **(F)** Simpson. **(G)** Pielou_e. **(H)** Unweighted PCoA. **(I)** Weighted PCoA.

To understand the mechanisms of action of AGBR in the gut microenvironment, the gut microbiota of patients with hyperlipidemia was analyzed using 16S rDNA sequencing. As shown in Figure 1A, 1,237 and 1,259 ASVs were detected in the AGBR and PR groups, respectively. A total of 638 ASVs overlapped between the two groups.

Subsequently, an alpha diversity analysis was performed. The goods_coverage was close to one in both groups, indicating sufficient sequencing depth (Figure 1B). The chao1 and observed_utos, which represent the microbial abundance, were both slightly higher in the AGBR group than in the PR group (Figures 1C,D). However, microbial diversity was slightly higher in the PR group than that in the AGBR group, with relatively higher Shannon and lower Simpson indices (Figures 1E,F). Additionally, the pielou_e, which represents microbial uniformity, was slightly higher in the AGBR group (Figure 1G). Microbial differences between the two groups were further analyzed using beta diversity. As shown in Figures 1H,I, a certain overlap and difference in microbial composition was observed between the AGBR and PR groups, either by weighted or unweighted PCoA.

### 3.5 Differential microbiota in patients with hyperlipidemia receiving AGBR or PR diets

**Figure 2:**
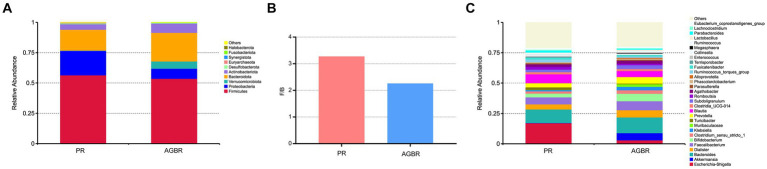
The differences of gut microbiota in patients with hyperlipidemia receiving AGBR or PR diets. **(A)** Relative abundances of top 10 microbial phyla. **(B)** F/B ratio. **(C)** Relative abundances of top 30 microbial genera.

Differences in the gut microbiota between the AGBR and PR groups were further analyzed. As shown in Figure 2A, *Firmicute*s, *Bacteroidota*, and *Proteobacteria* were the dominant phyla in the PR group, accounting for 56.47, 17.25, and 20.09%, respectively. Dietary intervention with AGBR decreased *Firmicute*s (53.51%) and *Proteobacteria* (8.41%); however, it elevated *Bacteroidota* (23.67%) in patients with hyperlipidemia. Some microbial phyla with relatively low abundance were also changed by the intervention of AGBR, such as *Verrucomicrobiota* (0.25% vs. 5.98%), *Desulfobacterota* (0.65% vs. 0.45%), and *Synergistota* (0.08% vs. 0.06%). Additionally, the ratio of *Firmicutes/Bacteroidetes* (F/B), an important indicator for the homeostasis of gut microbiota was lower in the AGBR group than in the PR group (Figure 2B).

**Figure 3:**
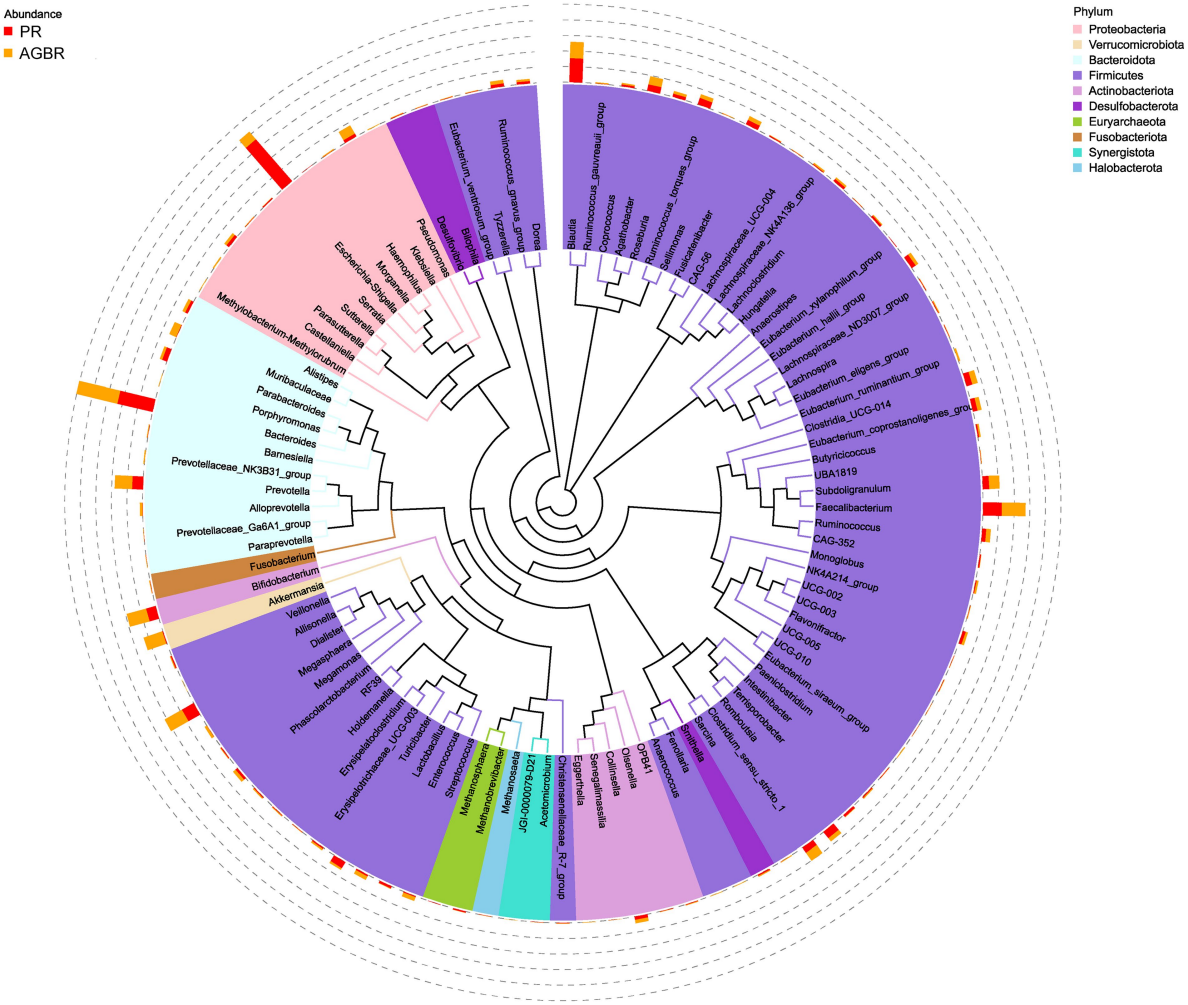
A tree representing the top 100 microbial genera in the top 10 microbial phyla in patients with hyperlipidemia receiving AGBR or PR diets.

At the genus level, *Escherichia-Shigella* was the most abundant in patients with hyperlipidemia; however, its abundance was reduced by AGBR intervention (17.05% vs. 2.98%). *Bacteroides*, another abundant microbial genus, was higher in the AGBR group than in the PR group (11.12% vs. 12.92%). In addition, AGBR elevated the abundances of *Faecalibacterium* (5.80% vs. 7.22%), *Dialister* (4.18% vs. 6.05%), *Prevotella* (3.33% vs. 5.34%), and *Bifidobacterium* (3.05% vs. 6.15%) in patients with hyperlipidemia. In contrast, AGBR reduced the abundances of *Blautia* (7.38% vs. 5.00%), *Romboutsia* (2.46% vs. 1.18%), and *Turicibacter* (2.17% vs. 0.51%) *in vivo* (Figure 2C). The top 100 microbial genera in the top 10 microbial phyla are shown in Figure 3.

## 4 Discussion

Hyperlipidemia is a prevalent metabolic disorder accompanied by the elevation of lipids in the blood (1). Hyperlipidemia seriously threatens human health because of the high risk of cardiovascular diseases (3). As diet is an important factor influencing hyperlipidemia, various functional foods have been developed to manage hyperlipidemia (22). GBR is a functional food rich in healthy components that are beneficial for the remission of human disorders, including hyperlipidemia (9). GBR inhibited high-fat diet-induced hyperlipidemia by improving lipid synthesis and metabolism in mice (10). In rat and rabbit models of hypercholesterolemia, GBR decreased TC and LDL-c levels and increased HDL-c levels (11, 12). However, research on the human body has been limited. In this study, the therapeutic potential of AGBR, a processed GBR, on hyperlipidemia was explored. Notably, dietary intervention with AGBR reduced TG, TC, LDL-c, and apolipoprotein-B levels and elevated HDL-c and apolipoprotein-A1 levels in patients with hyperlipidemia. These results are similar to those of previous studies on GBR in animal models, and illustrate that AGBR contributes to the remission of hyperlipidemia by affecting blood lipids.

Germination is an effective method for improving the sensory and nutritional qualities of brown rice. GBR is enriched in various nutritional components, including minerals, vitamins, dietary fibers, ferulic acid, γ-oryzanol, and γ-aminobutyric acid, which contribute to the health-promoting functions of GBR (9). Our previous study revealed that autoclaving further improves the taste of GBR and elevates the levels of GABA and ferulic acid (20). In this study, the nutritional characteristics of AGBR were analyzed. Free amino acids (10/16 species), resistant starch, soluble dietary fiber, and GABA were significantly higher in AGBR than in PR. The nutritional components in AGBR are similar to those in GBR and may be beneficial for the remission of hyperlipidemia through specific mechanisms. For example, resistant starch upregulates Acox1 (a lipid oxidation gene) and downregulates SREBP-1 (a fatty acid and triglyceride synthesis and metabolism-related gene) and Fads1 (a fatty acid synthesis gene) (23). Dietary fiber affects HMG-CoA reductase, LDL receptors, CYP7A1, MAPK signaling pathways, and other lipid metabolism-related target genes (24). GABA is involved in cholesterol metabolism in macrophages by blocking the p38MAPK and NF-κB signaling pathways (25). Notably, this study also found that AGBR was enriched in a considerable proportion of flavonoids (11/13 species). Flavonoids are a group of phytoestrogens with physiological activities in humans (26). Flavonoids have great potential to prevent and treat diverse metabolic disorders in humans, such as obesity, diabetes, hyperlipidemia, and osteoporosis (27). Some flavonoids that were detected to be elevated by AGBR in this study have also been demonstrated to be associated with the remission of hyperlipidemia, such as epicatechin (28), rutin (29), luteolin (30), quercetin (31), apigenin (32), and isorhamnetin (33). Therefore, flavonoids may be effective components of AGBR in the treatment of hyperlipidemia. However, the mechanisms underlying the effects of various flavonoids on hyperlipidemia are different and diverse and require systematic research and analysis.

The gut microbiota is a large and diverse group of microorganisms that play a crucial role in host metabolism and immune responses (34, 35). Evidence has shown that the gut microbiota is involved in the mechanisms of action of diverse crops, such as oats (4), tartary buckwheat (5), black rice (6), and red yeast rice (16), in alleviating hyperlipidemia. In this study, the therapeutic mechanisms of AGBR on the gut microbiota were explored. Compared with the PR group, patients with hyperlipidemia in the AGBR group exhibited relatively higher microbial abundance, diversity, and uniformity. These results indicate that AGBR may relieve hyperlipidemia by improving the gut microbiota.

At the phylum level, *Firmicutes* and *Bacteroidetes* are the main components of the human gut microbiota (36). The F/B ratio is regarded as an indicator of gut microbiota homeostasis and its elevation is associated with various metabolic syndromes, including obesity (37), hypertension (38), diabetes (39), and hyperlipidemia (40). Previous studies have revealed that many active substances with therapeutic potential against hyperlipidemia can reduce the F/B ratio, such as glycosaminoglycan (*Ostrea rivularis*) (41), polysaccharides (*Tremella fuciformis*) (42), edible kynurenic acid (*Tachypleus tridentatus*) (43), genistein (44), tomato seed oil (45), and highland barley whole grain (18). In this study, AGBR intervention decreased *Firmicute*s and elevated *Bacteroidota* in patients with hyperlipidemia, along with a decrease in the F/B ratio. These results are similar to previous findings and illustrate that the hypolipidemic effects of AGBR are associated with an increase in the F/B ratio. Additionally, AGBR intervention decreased *Proteobacteria*, *Desulfobacterota*, and *Synergistota*, and elevated *Verrucomicrobiota* in patients with hyperlipidemia. These microbial phyla may be involved in lipid homeostasis. As reported, *Proteobacteria* are more enriched in women with high total cholesterol levels than in those with normal serum lipid levels (46). Konjac glucomannan-dihydromyricetin reduces serum TG, total glycerol, and *Desulfobacterota* in mice fed a high-fat diet (47). *Synergistetes* are positively associated with cholesterol (48). *Lactobacillus sakei* MJM60958 inhibits lipid accumulation and increases *Verrucomicrobia* in mice with fatty liver (49). These microbial phyla may participate in the mechanism of action of AGBR in the treatment of hyperlipidemia.

The specific differences in the microbial composition between the PR and AGBR groups were further analyzed at the genus level. The results showed that *Escherichia-Shigella* and *Bacteroides* were the dominant genera, among which the former was reduced and the latter was elevated by AGBR intervention. *Escherichia-Shigella* is known as a harmful bacteria that accumulates in metabolic diseases (50). Li et al. reported that *Spirulina platensis* polysaccharides reduces *Escherichia-Shigella* and improves lipid and carbohydrate metabolism in rats fed a high-fat diet (51). *Bacteroides*, belonging to *Bacteroidetes*, is a beneficial bacteria associated with metabolic diseases (52). Previous studies have revealed that an increase in *Bacteroides* is associated with remission of hyperlipidemia in rats (53, 54). Changes in these two genera may have contributed to the hypolipidemic effects of AGBR *in vivo*. In addition, this study also found that AGBR elevated *Faecalibacterium*, *Dialister*, *Prevotella*, and *Bifidobacterium*; however, it reduced *Blautia*, *Romboutsia*, and *Turicibacter* in patients with hyperlipidemia. These microbial genera may also participate in the therapeutic mechanisms of GBR on hyperlipidemia through regulating lipid homeostasis (4, 52, 55–60).

However, this study has some limitations. For example, the sample size was relatively small and the intervention time was relatively short. The relationship between the nutritional components of AGBR, especially flavonoids, and blood lipids, has not been thoroughly studied. Additionally, the specific effects and mechanisms of action of different bacteria on blood lipid levels remain unclear. Further studies on these fields are warranted in the future.

## 5 Conclusion

In conclusion, dietary intervention with AGBR reduced blood lipid levels in patients with hyperlipidemia. AGBR is rich in free amino acids, resistant starch, soluble dietary fibers, GABA, and flavonoids. Additionally, AGBR improved the gut microbiota in patients with hyperlipidemia, evidenced by reduced *Firmicutes*, *Proteobacteria*, *Desulfobacterota*, and *Synergistota*, and elevated *Bacteroidota* and *Verrucomicrobiota* at the phylum level, as well as reduced *Escherichia-Shigella*, *Blautia*, *Romboutsia*, and *Turicibacter*, and elevated *Bacteroides*, *Faecalibacterium*, *Dialister*, *Prevotella*, and *Bifidobacterium* at the genus level. AGBR may be an effective functional food to relieve hyperlipidemia by modulating the gut microbiota.

## Data availability statement

The data presented in this study are deposited in the SRA repository, accession number: PRJNA1107690.

## Ethics statement

The studies involving humans were approved by Lianshui People’s Hospital affiliated to Kangda College, Nanjing Medical University. The studies were conducted in accordance with the local legislation and institutional requirements. The participants provided their written informed consent to participate in this study.

## Author contributions

CR: Conceptualization, Data curation, Formal analysis, Funding acquisition, Visualization, Writing – original draft. BH: Data curation, Formal analysis, Writing – original draft. SZ: Data curation, Formal analysis, Writing – original draft. DY: Data curation, Investigation, Writing – original draft. JF: Data curation, Investigation, Writing – original draft. SS: Data curation, Investigation, Writing – original draft. JZ: Data curation, Investigation, Writing – original draft. LG: Data curation, Investigation, Writing – original draft. LZ: Data curation, Investigation, Writing – original draft. SL: Conceptualization, Funding acquisition, Supervision, Writing – review & editing.
